# Prevalence of Heavy Alcohol Use by Caregivers of Persons With Alzheimer’s Disease or Related Dementia

**DOI:** 10.1093/geroni/igaa057.1154

**Published:** 2020-12-16

**Authors:** Zachary Kunicki, Richard Jones

**Affiliations:** Brown University, Providence, Rhode Island, United States

## Abstract

Some caregivers of persons with Alzheimer’s Disease and related dementias (ADRD) are known to be under high levels of burden, which is associated with higher levels of anxiety, depression, and stress. Previous research has established anxiety, depression, and stress are associated with heavy alcohol use, but little research has examined heavy alcohol use among ADRD caregivers. Heavy alcohol use could influence the ability of ADRD caregivers to provide care. The purpose of this study was to explore the prevalence and prevalence correlates of heavy alcohol use among ADRD caregivers using the 2016 Behavior Risk Factor Surveillance Survey (BRFSS). We identified 2,028 persons among the 486,303 BRFSS respondents who were the primary informal caregivers of a person with ADRD. Among them, the prevalence of heavy alcohol use was 6.3 per 100 persons. Adult child caregiver relationship, positive smoking status, and fewer hours of providing care per day were all positively and significantly associated with heavy alcohol use. Notably, sex was not. Future research should examine if heavy alcohol use by ADRD caregivers is related to personally and clinically relevant outcomes of care provided to the persons with ADRD.

